# Methods of Cryoprotectant Preservation: Allogeneic Cellular Bone Grafts and Potential Effects

**DOI:** 10.1155/2019/5025398

**Published:** 2019-10-16

**Authors:** W. Blake Martin, Renaud Sicard, Shabnam M. Namin, Timothy Ganey

**Affiliations:** ^1^Vivex Biomedical, Inc., Atlanta, GA, USA; ^2^Vivex Biomedical, Inc., Miami, FL, USA

## Abstract

Debridement of the bone surface during a surgical fusion procedure initiates an injury response promoting a healing cascade of molecular mediators released over time. Autologous grafts offer natural scaffolding to fill the bone void and to provide local bone cells. Commercial bone grafting products such as allografts, synthetic bone mineral products, etc., are used to supplement or to replace autologous grafts by supporting osteoinductivity, osteoconductivity, and osteogenesis at the surgical site. To assure osteogenic potential, preservation of allogeneic cells with cryoprotectants has been developed to allow for long-term storage and thus delivery of viable bone cells to the surgical site. Dimethyl sulfoxide (DMSO) is an intracellular cryoprotectant commonly used because it provides good viability of the cells post-thaw. However, there is known cytotoxicity reported for DMSO when cells are stored above cryogenic temperatures. For most cellular bone graft products, the cryoprotectant is incorporated with the cells into the other mineralized bone and demineralized bone components. During thawing, the DMSO may not be sufficiently removed from allograft products compared to its use in a cell suspension where removal by washing and centrifugation is available. Therefore, both the allogeneic cell types in the bone grafting product and the local cell types at the bone grafting site could be affected as cytotoxicity varies by cell type and by DMSO content according to reported studies. Overcoming cytotoxicity may be an additional challenge in the formation of bone at a wound or surgical site. Other extracellular cryoprotectants have been explored as alternatives to DMSO which preserve without entering the cell membrane, thereby providing good cellular viability post-thaw and might abrogate the cytotoxicity concerns.

## 1. Introduction

Most surgical bone grafting procedures involve additional disruption or injury of the local bone at the surgical site, such as the roughening of the end plates for interbody fusions, abrading the transverse processes, removal of the spinous processes for a posterolateral spine fusion, or the scratching of the bone joints in a foot and ankle surgical procedure. In all cases described, the intention is to remove tissue such as cartilage or fibrocartilage that is inherently avascular, which might prevent adequate access to a bleeding vascular source. The bone repair process occurs as a response to injury [1–6]. Giannoudis et al. described it well as a “well-orchestrated, biological phenomenon involving the interaction of molecular mediators and cellular elements” [3]. For indirect bone healing, or the most common way that bone heals, a hematoma forms during an acute inflammation response, then mesenchymal stem cells are recruited that leads to the formation of a cartilaginous callus which preserves volume, and allows for the emergence of an osseous type matrix [1–6]. The fracture site is revascularized, and the callus is mineralized followed by final remodeling [1–6]. Mirroring the stages of hematoma, mesenchymal recruitment, and bone formation through additional debridement has become a part of bone grafting procedures.

## 2. Inflammation Stage of Bone Healing

The acute inflammation response is key to the overall healing cascade as it peaks at 24 hours and is complete after 7 days [4–6]. What are the primary actors in this inflammation response? They are neutrophils, lymphocytes, and macrophages [3]. These cells release a host of cytokines, or cell signaling molecules, thus triggering further chemotaxis of inflammatory cells, encouraging extracellular matrix synthesis, stimulating angiogenesis, and bringing fibrogenic cells to the injury site [3]. Within 24 hours after injury, the cytokine production peaks, but will increase again during bone remodeling [5]. Specific inflammatory cytokines that lead to bone formation are interleukin-1a (IL-1a), IL-1b, IL-6, IL-11, IL-18, cyclooxygenase (Cox2), macrophage colony-stimulating factor (MCSF), and tumor necrosis factor alpha (TNF-*α*) [1, 3]. It is a properly balanced inflammatory process that leads to bone healing as overactive inflammation, perhaps as a response to infection, may inhibit the bone repair process [3]. Also, other conditions, such as diabetes, may lead to delayed bone healing [7].

## 3. Common Bone Grafting Products

**Figure 1:**
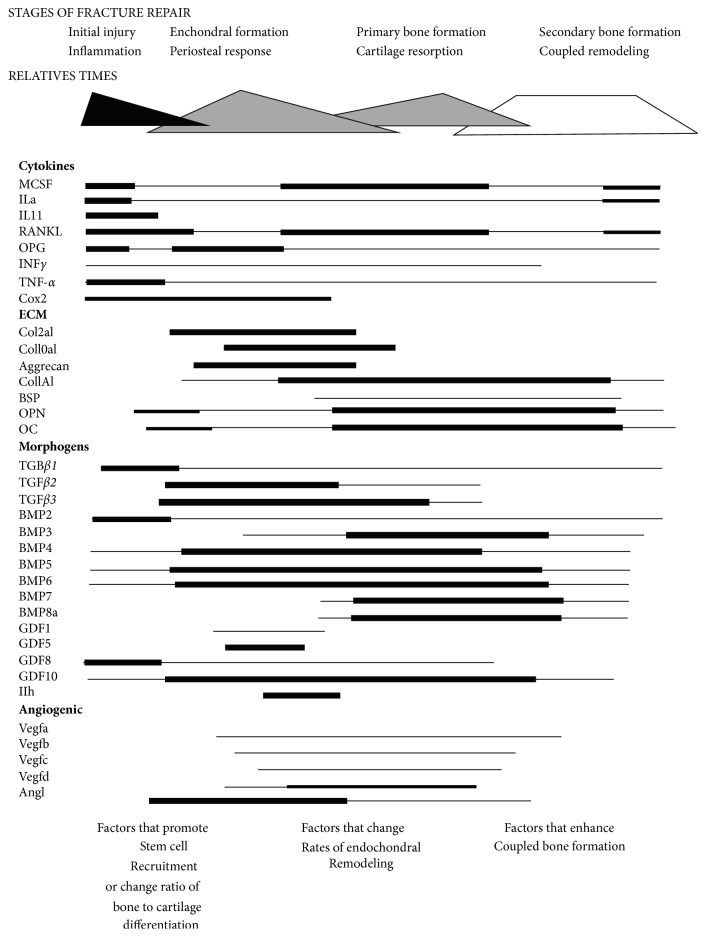
Schematic summary of stages of fracture repair and their associated molecular processes. The relative temporal aspects of each of the stages of the fracture healing process are denoted by basic geometric shapes that also connote the relative intensity of the molecular processes that define each of the stages. The levels of expression are by percent over baseline for each and are not comparable between individual mRNAs. Time frames and strategies to alter repair at various stages are indicated at the bottom of the figure. Reprint from Gerstenfeld and Einhorn [1].

To assist in the bone healing response, most surgeons will include bone grafting products. Surgeons take into consideration the type of procedure, comorbidities, and the expected osteogenic capacity of the patient to determine the types of bone grafting products that are needed. The historical “Gold Standard,” or autologous graft, consists of mineralized bone tissue, typically cancellous bone that facilitates transfer of local bone cells. Autologous graft provides enough natural scaffolding to fill the bone void or grafting site, and supplementary cellular material to encourage the bone healing response. However, obtaining autologous graft, often harvested as healthy bone from the iliac crest, may lead to graft site morbidity and undesirable side effects such as postoperative pain, chronic pain, infection, scarring, etc. Thus, alternatives to autologous grafts have been used by surgeons for the past several decades. Examples of scaffold products include cortical/cancellous bone chips, synthetic bone mineral products, collagen, etc. In addition, morphogens have been described as important factors in the bone healing cascade [1]. Defining bone morphogeneic proteins (BMPs) within bone grafting products, such as with demineralized bone matrix (DBM), is now common in commercial products [8]. Gerstenfeld and Einhorn provided an excellent overview revealing the effects of the network of BMPs during the bone healing cascade as well as the effects of the cytokines, extracellular matrix, and angiogenic growth factors (Figure 1) [1]. More recently, allogeneic cells have been included in commercial bone allograft products as the cells may assist at the bone grafting site. The bone healing cascade is a multi-factorial process and each of these bone grafting products may assist in various stages of that response [1].

To optimize handling of bone grafting products or preserve the allogeneic cells, additional materials are typically included. For DBM products, carrier materials such as carboxymethylcellulose (CMC), glycerol, hyaluronic acid, or porcine gelatin may be included to provide better handling characteristics for the surgeon. For allogeneic cell preservation, a cryoprotectant is required so that the cells may survive the freeze and thaw process [9–14]. Each of these additional materials may also influence the bone grafting site.

## 4. Cryoprotectants

The purpose of cryopreservation is to maintain cell viability at extremely low temperatures for long-term storage and transport; such that the cells may be thawed at the time of surgery and provide benefits at the bone grafting site [9]. Cryoprotectants may generally be classified as either intracellular or extracellular, which describes their mechanism of action as to whether they penetrate the cell membrane or not [9]. Examples of intracellular cryoprotectants are dimethyl sulfoxide (DMSO), glycerol, formamide, and 1,2-propanediol, and examples of extracellular cryoprotectants are large weight polymers, polypeptides, sugars, polyvinyl pyrrolidone, and poly (ethylene glycol) [9–11]. It is necessary that the addition and removal of the cryoprotectants is controlled to preserve cell viability, cell differentiation, and cell signaling [9–14]. A slow freezing process of about -1 °C/min is commonly used for freezing so that most of the intracellular water has enough time to diffuse into the extracellular space prior to ice crystal formation [11–13]. Freezing too quickly will result in more ice formation within the cells and lead to more cell death [11–13]. For intracellular cryoprotectants, freezing too slow results in long-term exposure to high intracellular cryoprotectant concentrations as the cells are dehydrated and experience severe volume shrinkage [11–13]. For extracellular cryoprotectants, the cells are coated and may improve the osmotic imbalance when compared to intracellular cryoprotectants [15].

DMSO is an intracellular cryoprotectant extensively used in the research laboratory as it provides good cellular viability so long as the cryoprotectant can be quickly removed post-thaw. Recognizing that long-term exposure to DMSO has been shown to be cytotoxic at room temperatures, typical laboratory protocols for a removal process involve dilution, centrifugation, removal of all the media separate from the cellular pellet, replacement with fresh media, and resuspension of the cellular pellet [9–15].

**Figure 2:**
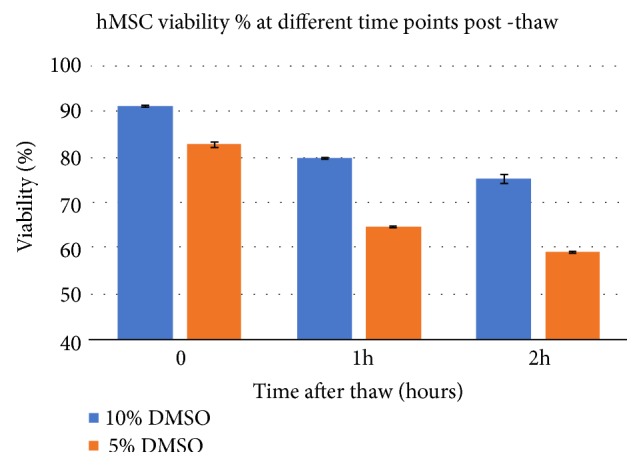
Decreased viability of hMSCs within 2 hours post-thaw at ambient temperatures followed by 48-hour culture and then an alamar blue assay.

In contrast, allogeneic cells within a cellular bone graft product are typically attached within the matrix of the mineralized bone. Thus, most of these products can only rely on washing or decanting of the extra DMSO prior to implanting, as centrifugation is clearly not an option. Regardless, residual amounts of DMSO may remain even after washing or decanting as DMSO is an intracellular cryoprotectant. More recent allogeneic cellular bone grafts aim to provide a minimal amount of DMSO cryoprotectant such that the bone graft may not be oversaturated. These tissue providers suggest that their product be thawed but not washed nor decanted prior to implanting, which likely leaves even more DMSO to be implanted. The percentage of DMSO varies from one tissue supplier to another but typically ranges from 10% to not less than 5% as fewer cells will survive the freezing process for smaller DMSO percentages (Figure 2) [16].

## 5. DMSO Cytotoxicity

DMSO has been well studied as it relates to its cytotoxicity and osmotic shock to cells [9–15]. However, much of the focus of commercial allogeneic cellular bone grafts has remained on the cell viability immediately post-thaw and the presence of certain cell types [17–20]. Since the removal of the cryoprotectant strays from the centrifugation process used in the research laboratory, the effects of residual DMSO implanted into the bone grafting site is less known. Relatively little focus has been directed toward its intracellular effects at body temperature [13, 16, 21]. Eventually, the DMSO may be expunged from within the cells into the grafting site as water replaces the DMSO. However, the DMSO may then affect the host cells present at the grafting site. One of the key assets of the hemodynamic process attributable to bone injury is the walling off of the injury site with a hematoma, or blood clot. One of the less considered results of bone fracture, or wounding in general, is the disruption in circulation.

Where arterial supply and venous return once nourished tissues interconnected through capillaries, the disruption and clot formation change the perfusion, oxygen tension, cell deposition, etc. Neither hypoxia nor hyperoxia significantly alter chondrogenesis or osteogenesis during early stages of fracture healing, and macrophage and neutrophil infiltration are unaffected by environmental oxygen after bone injury [22]. Perhaps accepted as intuitive, this seminal vascular disassociation serves several biologic functions; first stopping the blood flow, second reducing the potential for emboli to be transferred during the clotting process, and third defining a consolidated volume of tissue that will subsequently remodel via acknowledged regenerative and reparative methods. Less intuitive and more pertinent to this discussion is the local stasis of the cryoprotectant following delivery in cells. The argument might be made that cubic centimeters of tissue will be so vastly diluted by the volume of peripheral circulation that the toxicity of the cryoprotectant would be immaterial. Given the intention to replace a bone void, or augment natural processes, delivery of the viable allograft into a wound site does not connect through the peripheral circulation and is not exposed to wash out. Moreover, the toxicity in diffusion after placement has direct contact with host tissue that wounded is receiving immense numbers of cytokine signals. Cell death and apoptosis do not seem to harbor biopotential although the full understanding remains to be elucidated. While some clinical success of allogeneic cellular bone grafts has been reported, cytotoxicity effects may be present which need to be overcome and may depend in part on the osteogenic capacity of the patient to do so [23–28]. One review of cryopreservation studies reported that while mesenchymal stromal cells (MSCs) “have proved amenable to cryopreservation by conventional slow cooling protocols, their pluripotent counterparts have been shown to be more refractory” [16]. Reubinoff et al. reported 16% recovery of pluripotent cells with 10% DMSO after freezing and thawing [29]. Also, Kloverpris et al. studied human peripheral blood mononuclear cells and determined that “the exposure time to DMSO can be more harmful than the concentration itself” as 0.2% DMSO over time had more effect than 10% DMSO for one hour [30].

Recalling that the early bone healing response includes a hematoma formation, DMSO has been studied relating to the toxic effects on red blood cells (RBCs), platelets, and vascular endothelial cells [4–6, 31]. DMSO was toxic at 0.2%–0.4% for RBCs in vitro as DMSO changed the RBCs internal and external material imbalance [31]. DMSO was also toxic to vascular endothelial cells at 0.6%, and inhibited platelet aggregation in a dose dependent manner from 0.25% to 6% DMSO [31]. In addition, DMSO has been shown to destabilize some proteins and change the apparent binding properties even at low concentrations [32].

Another important characteristic of early bone healing is the presence of key cytokines as DMSO may influence lymphocyte activity and have anti-inflammatory effects [1, 4–6, 33, 34]. Costa et al. reported that the cell viability of peripheral blood mononuclear cells (PBMC) were not reduced for 2.5% DMSO in-vitro but was for 5% and 10% DMSO [33]. However, 1%–2% DMSO was found to reduce the cellular proliferation and decreased the cytokine production, particularly for TNF-*α* [34]. TNF-*α* is a cytokine in the early inflammation stage of bone healing [1, 3–6, 30]. Costa et al. concluded that the effects were less cell specific but instead were focused on the overall reduction in cell signaling [34]. Reduced cell signaling may delay the bone grafting process as the implanted allogeneic cells and the local cells recover from the residual DMSO that was also implanted.

**Figure 3:**
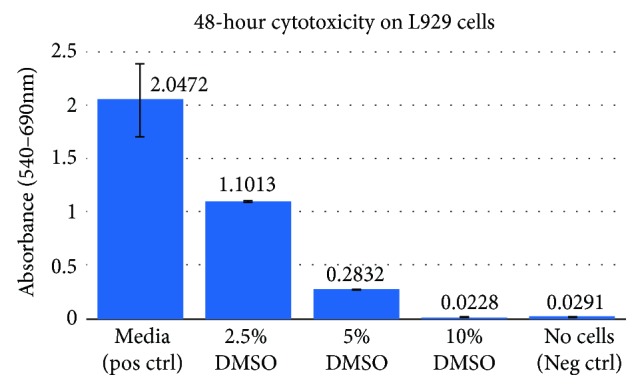
48-hour cytotoxicity results performed on L929 cells EMEM/10% FBS (positive control), 2.5% DMSO, 5% DMSO, 10% DMSO, and no cells (negative control).

Fibroblasts are important for the early extracellular matrix process during early bone formation [1, 35]. Using a neutral red uptake assay, DMSO media samples of different concentrations were prepared. DMSO was determined to have a cytotoxic effect based on the ability to maintain L929 cell viability after 48-hour exposure compared to positive control conditions (Figure 3). While 10%–5% is reported as necessary as the percentage DMSO in allogeneic cellular bone grafts to preserve enough cells during the freezing process, 2.5% DMSO was also studied as a comparison [16]. Other studies have reported that exposing fibroblasts to even 2% DMSO caused alterations in cell morphology [36].

## 6. Extracellular Alternatives

Recently, researchers began to focus more attention on extracellular cryoprotectants as alternatives to DMSO as they do not present similar cytotoxicity effects perhaps in part as the cellular membrane is not permeated by the cryoprotectant [15, 21]. In one study, human amniotic fluid stem cells were investigated with cryopreservation via both intracellular and extracellular agents [15]. Cell survivability overall remained greater for the intracellular, DMSO cryopreservation than extracellular, disaccharide cryopreservation utilizing either sucrose or trehalose [15]. However, the cellular viability of DMSO and glycerol was lower at six months of storage versus three months while the sugar-based cellular viability was higher at six months [15]. As more research is focused on the effects of either the residual DMSO or the implanted DMSO in the context of allogeneic cellular bone graft products, more extracellular cryoprotectants will continue to be explored. Halford recently reported on carbohydrate-based cryoprotectants and polymer based cryoprotectants as other extracellular options [37]. Could an extracellular cryoprotectant provide high cell viability postthaw without the cytotoxicity effects of a DMSO cryoprotectant? Could an extracellular cryoprotectant provide better preservation of the cellular material within the allogeneic cellular bone graft and have less of a potential effect on the host bone grafting environment?

A better understanding is achieved with a mechanistic consideration of what value extracellular protection affords. More than viability, the enhancement affords pericellular gradients that enhance communication between cells, matrix, and other cells. Protection in scope serves dual purposes; insulating from ice crystal microfracture the obvious, but also sustaining a permissive electrochemical barrier that enhances exchange without compromising protection. One of the early observations aligned to freeze concentration recognized a physicochemical phenomenon wherein water molecules crystallize to form ice, leading to increased solute concentrations in the remaining unfrozen solution forming a phase separation during freezing [38].

More specifically, it is known that during ice nucleation that ice grows in all directions when a solution is supercooled at −5 to −45°C. During the process of the freezing, high solute concentration remains in the unfrozen solution leading to a concentrated solute around the cells located in the residual solution [39]. By calculating the sodium ion concentration during freezing in the presence of a cryoprotectant, the amount of residual water can be measured by 1H-NMR [40]. Data from this technology discern a difference in sodium concentration using DMSO compared to a polyampholyte cryoprotectant as an extracellular alternative. At the surface of cells protected by DMSO (intracellular saturation) at −40°C, the sodium ion concentration is approximately 7 times higher than it was before freezing. With polyampholyte as a cryoprotectant, the sodium ion is concentrated >10 times higher than that at room temperature, indicating that the extracellular concentration of certain materials increases because they are ejected from ice crystals during freezing. Other reports have offered a sophisticated treatment of the permittivity of crystallization potentiated by dipole modelling [41]. Invigorated by data demonstrating DMSO, which has an oxygen atom with a strong negative charge and a sulphur atom with a strong positive charge, the influence of this high dipole moment strongly affects the water freezing process, interceding in reduced crystal size to protect fractionally small moments of hysteresis and protecting, by size restriction and freeze/thaw excursions, the adjacent cell membranes [42].

This phenomenon offers an exceptional strategy for an extracellular cryoprotectant to enhance adsorption of protein/carrier complexes adjacent to cells, owing to the increase in the peripheral cell concentration of the excluded fractions during the freezing process. With context of cell biologics and preservation, the somewhat elevated difference in ionic and protein complexes captured in the pericellular space affords an asset to equilibrium, providing cell-actuated exchange outside rather than inside, or at the membrane. The complexity of the effect nonwithstanding, retention of cell viability and pericellular dilution also behave as active reservoirs of the conditions to which they were preserved. While still a significant amount to be known in this field, offering an encompassed entity sustainable placement, marked gradients during the thaw, and reaction efficiencies in both dilution and osmotic exchange, cells are somewhat tidal in the sense that the excursion of ions, proteins and ligands will flow from the cell. Within this strategy, the interaction between the cell membrane and protein/carrier complex is distinguished by directional or vectored viability from its initial circumferential concentration.

Different advantages are afforded by the complementary logic of delivery. When placed into an injury bed for bone repair, the adsorbed complexes of the wound fluid are buffered rapidly at the interface and normalized, if not internalized instead of diffusing back into the solution. This suggests a possibility for pharmacologic reduction, and titering quantity of valuable materials needed for internalized delivery into cells. In the context of extracellular protection, freeze concentration strategies offer several distinct advantages in that they are simple, cost-effective, highly reliable, and characterized by a lack of toxicity, high cell viability, and enhanced interaction between host growth factors and the cell membrane.

## 7. Conclusion

Bone carries an amazing capacity for self-repair that adapts throughout life to the subtleties of mechanical stimulation. Sometimes mechanical forces exceed the material limits, and when that happens bone fractures. Processes incumbent to repair emerge from the milieu at the site of injury to consolidate the repair and reintegrate. However, for the injuries where a large mass of bone is lost through disease, surgical incision, or traumatic insult, grafting materials were developed to supplement the critical sized defect, and support the necessary stages of osteoinductivity, osteoconductivity, and osteogenic activity. This third feature of grafting materials relies on cell activity to actively guide differentiation of uncommitted cells to an osteogenic or bone-based cell lineage. The advent of viable cell allografts has made this possible.

As viable allografts emerged as an addition to the armamentarium for treating bone voids, a parallel technology for supporting cell viability was needed as well. With key constraints on functional benefits of preserving without modifying, avoiding toxicity to cells during thaw, and enhancing therapeutic performance, cryoprotectants became an established foundation. Reducing ice crystallization, suppressing crack propagation, and demonstrating phenotypic durability are common assets. As noted in this discussion, potential postthaw toxicity for DMSO, the commonly used intracellular cryoprotectant, not only effects the cellular component of viable graft material, but also harbors risk for the adjacent tissue in the host which is being treated. Recent data have demonstrated evidence that viability with extracellular protectants are on par during the freeze period with intracellular protectants. Extracellular cryoprotectants are gaining more interest in order to reduce the risks of intracellular cryoprotectant-related toxicity.

Clinical challenges concomitant to tissue loss have benefitted from viable allograft in surgical fusions [43]. Moreover, with low complications, high regenerative potential, and economic value in reduced time to healing, higher rate of fusion, and shorter windows of recovery, advances in cryoprotectant technology will continue to improve. Allograft has been among the most essential of surgical tools available to orthopaedic surgeons, and as nonpharmacologic enhancement with viable allograft integrates the operating theater, assurances that maximal biologic function is maintained in delivery is critical.

## Data Availability

The hMSC viability data used to support the findings of this study are available from the corresponding author upon request. The L929 cytotoxicity data used to support the findings of this study are available from the corresponding author upon request.
